# Microbial Primer: The catalytic biofilm matrix

**DOI:** 10.1099/mic.0.001497

**Published:** 2024-08-30

**Authors:** Lise Goltermann, Shahab Shahryari, Morten Rybtke, Tim Tolker-Nielsen

**Affiliations:** 1 Costerton Biofilm Center, Department of Immunology and Microbiology, University of Copenhagen, DK-2200, Copenhagen, Denmark

**Keywords:** biofilm, catalytic functions, environment, extracellular polymeric substances, infections, matrix

## Abstract

The extracellular matrix of microbial biofilms has traditionally been viewed as a structural scaffold that retains the resident bacteria in the biofilm. Moreover, a role of the matrix in the tolerance of biofilms to antimicrobials and environmental stressors was recognized early in biofilm research. However, as research progressed it became apparent that the biofilm matrix can also be involved in processes such as bacterial migration, genetic exchange, ion capture and signalling. More recently, evidence has accumulated that the biofilm matrix can also have catalytic functions. Here we review foundational research on this fascinating catalytic role of the biofilm matrix.

## Introduction

Microbial biofilms are aggregates of micro-organisms that are embedded in a matrix of extracellular polymeric substances [1]. The aggregates can appear as microcolonies attached to a surface or as non-attached entities [2]. Biofilms are ubiquitous in the environment, and are indispensable as they are the main drivers of biogeochemical cycling processes of the elements [1]. They significantly impact various areas of society, ranging from beneficial roles in wastewater treatment and biofuel production to detrimental biofouling of industrial plants and ships’ hulls.

Biofilms are also present inside humans and animals, where most of these microbes constitute the normal microbiome of the host. However, in some cases they can be the cause of infections. Biofilm infections are problematic, as bacteria in the biofilm mode of growth display tolerance to immune system activities and the current assortment of antibiotics [3]. As a result, biofilms are a common cause of persistent infections, such as those affecting lungs, urinary tracts, ears and wounds [3]. They are also responsible for persistent infections associated with implanted medical devices and catheters [3].

The extracellular matrix of biofilms is primarily produced by the microbes themselves, although components from the environment or infectious sites in some cases can be incorporated. The composition of the biofilm matrix is highly diverse among microbial species, but generally, the main components are polysaccharides, extracellular DNA (eDNA), lipid vesicles, and proteinaceous factors such as pili, amyloid fibres, lectins and adhesins [1]. The matrix is primarily thought to function as the scaffold of biofilms and a shield against antimicrobials and environmental insult [1]. However, the biofilm matrix can also be involved in processes such as bacterial migration, genetic exchange, ion capture and signalling [4]. More recently, evidence has emerged that the biofilm matrix can also have catalytic functions. In the present Microbial Primer, we focus on the catalytic functions of the biofilm matrix, as increasing evidence suggests that this is an important feature in both environmental and medical settings.

## Catalytic functions of the matrix of environmental biofilms

**Fig. 1.**
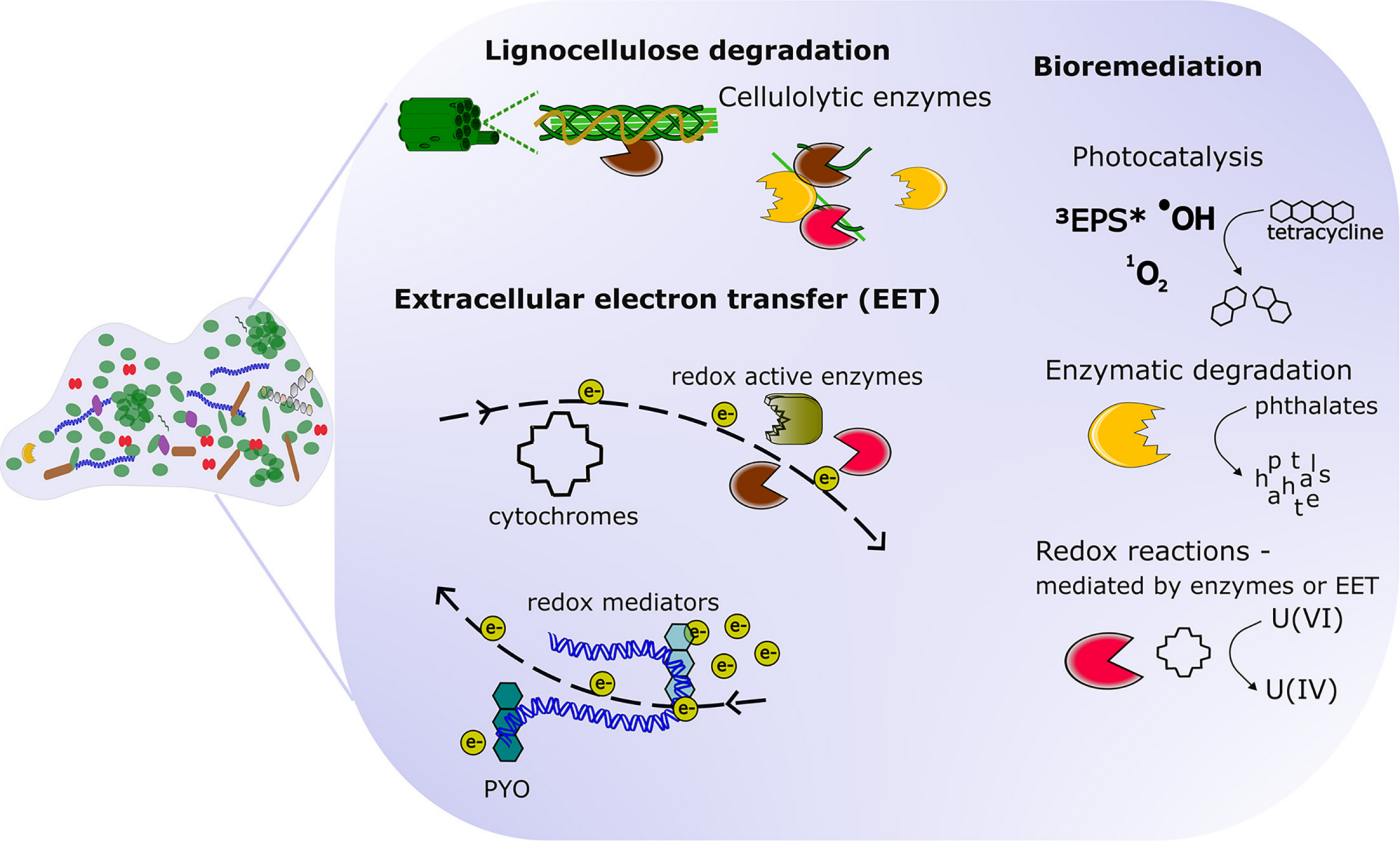
Catalytic properties of the extracellular matrix play a role in environmental and biotechnological biofilms. The extracellular matrix of environmental biofilms contains secreted hydrolases and hydrolase complexes capable of breaking down lignocellulose to facilitate carbon cycling. Extracellular electron transport in the matrix is mediated by redox active proteins, cytochromes, eDNA, pili, phenazines and other small molecules, and facilitates anaerobic respiration and plays a role in biotechnological applications (e.g. bioelectrochemical systems). Pollutants can be degraded by matrix-associated enzymes, or undergo redox modification mediated by redox active enzymes or charge transfer through the matrix, such as the reduction of water-soluble uranium(VI) into insoluble uranium(IV). Photocatalysis can facilitate pollutant breakdown through the action of reactive oxygen species generated in the matrix.

Biofilms are found on most surfaces in natural environments, where the extracellular matrix and the general architecture of mono- and multispecies biofilms allow the capture and retention of both dissolved and particulate matter from their surroundings. Environmental biofilms play a key role in biogeochemical processes, significantly contributing to the cycling of various elements, such as carbon and nitrogen. The catalytic functions of the extracellular matrix of microbial biofilms are multifaceted and play a crucial role in various environmental and biotechnological processes (Fig. 1).

Plant biomass, which is composed of lignocellulose consisting of cellulose, hemicellulose and lignin, is highly abundant in aerobic and anaerobic ecosystems, and a set of specialized enzymes are required to achieve its complete degradation. Under aerobic conditions in soil, streams and rivers, biofilms consisting of prokaryotic and eukaryotic micro-organisms are present on practically every available surface. The biofilm matrix retains and protects extracellular cellulolytic enzymes, which work together to break down carbon-rich polymeric plant fibres in an extracellular digestion system. These enzymes, which can be found solitary or in complexes, mainly consist of glycoside hydrolases, i.e. endoglucanases, exoglucanases and cellobiases [5].

Under anaerobic conditions, molecular oxygen is not available to function as the terminal electron acceptor, and the biofilm matrix becomes important in facilitating extracellular electron transport, which is vital for various metabolic processes. A significant niche for anaerobic lignocellulose degradation is the rumen of ruminant animals, where biofilms consisting of multiple microbial species form so-called syntrophic consortia. Extracellular electron transport is essential in these consortia, where electrons via the biofilm matrix are shuttled between species to facilitate metabolic coupling between biofilm members. Fermentative bacteria can transfer electrons either indirectly via H_2_ or formate or directly through interspecies electron transfer mechanisms to neighbouring methanogens, resulting in methane production [5]. Extracellular electron transport also allows species such as *Geobacter* and *Shewanella* to use insoluble or solid-state electron donors and acceptors such as iron or manganese-containing minerals. This capability is essential for microbe-mediated element cycling under anaerobic conditions, such as those found in marine sediments. In *Geobacter* spp. biofilms, this long-range electron transport is facilitated by extracellular electricity-conducting filaments, known as nanowires, which are part of the biofilm matrix and consist of pili structures and extracellular cytochromes OmcS and OmcZ. In addition, extracellular electron transport can be supported by redox-active enzymes or small molecules that can diffuse in the matrix, such as matrix-associated iron in the case of *Enterococcus faecalis*, riboflavin in the case of *Shewanella oneidensis* and phenazines in the case of *Pseudomonas aeruginosa*. Recent research suggests that the eDNA of the matrix also aids in charge transfer [6].

The mechanisms that make biofilm communities an integral part of the global biogeochemical cycles also contribute to biofouling and biocorrosion when contaminating biofilms colonize surfaces in production facilities or infrastructure. For example, redox active enzymes released into the biofilm matrix may be a contributing factor to the microbe-influenced corrosion of iron, posing a significant problem to iron and steel installations (reviewed by Conners *et al*. [7]).

Increasing understanding of the catalytic properties of the biofilm matrix means its abilities can be harnessed for biotechnological applications. The biofilm matrix is gaining attention for its potential use in bioremediation and bioproduction. Especially in environments subjected to wastewater, such as soil, rivers and lakes, the extracellular matrix from the resident microbes has been shown to contribute to pollutant degradation. This can occur through direct enzymatic activity via matrix-associated hydrolases or oxidoreductases, as demonstrated in the degradation of phthalate esters by soil and aquatic species such as *Rhodococcus* sp. and *Gordonia* sp [8].

Breakdown of organic pollutants can also be achieved through photodegradation mediated by the biofilm matrix. Upon UV light excitation, the matrix is capable of producing oxygen radicals such as singlet oxygen and hydroxyl radicals or a triplet excited state of the matrix itself. These light-generated reactive oxygen species were shown to facilitate the degradation of pollutants such as tetracycline, rhodamine and sulfadiazine [8].

Furthermore, matrix-mediated electron transfer of electroactive biofilms may also aid in removal of organic and inorganic environmental pollutants. Using the electrochemical properties of specific biofilm species, systems can be designed specifically for targeted pollutant removal. For example, soluble uranium(VI) can be removed by reduction into insoluble uranium(IV) by *Geobacter* species receiving electrons either from organic compounds or a supplied electrode [9].

Electrochemical systems based on microbial biofilms, in which induced cell lysis is a part of their development process, can take advantage of the lytic release of redox-active enzymes or the secretion of extracellular electron transport mediators into the surrounding biofilm matrix and their cumulative sorption to an electrode surface. Whether extracellular electron transport occurs via direct or indirect methods, electroactive biofilms can provide a scaffold for microbial fuel cells, where cellular metabolism is coupled to external electron transport to produce an electrical current. Electron transport in the matrix of biofilms can also play a role in bioelectrosynthesis systems. A promising example is the bioelectrosynthetic conversion of CO_2_ into value-added products such as acetate (reviewed by Conners *et al*. [7]). Acetogens and methanogens can convert CO_2_ into acetate and methane, respectively, by accepting electrons from a supplied cathode. Microbial electrosynthesis systems consisting of microbial consortia with coupled metabolisms may also be designed for the production of more complex end-products.

## Catalytic functions of the matrix of medically relevant biofilms

**Fig. 2.**
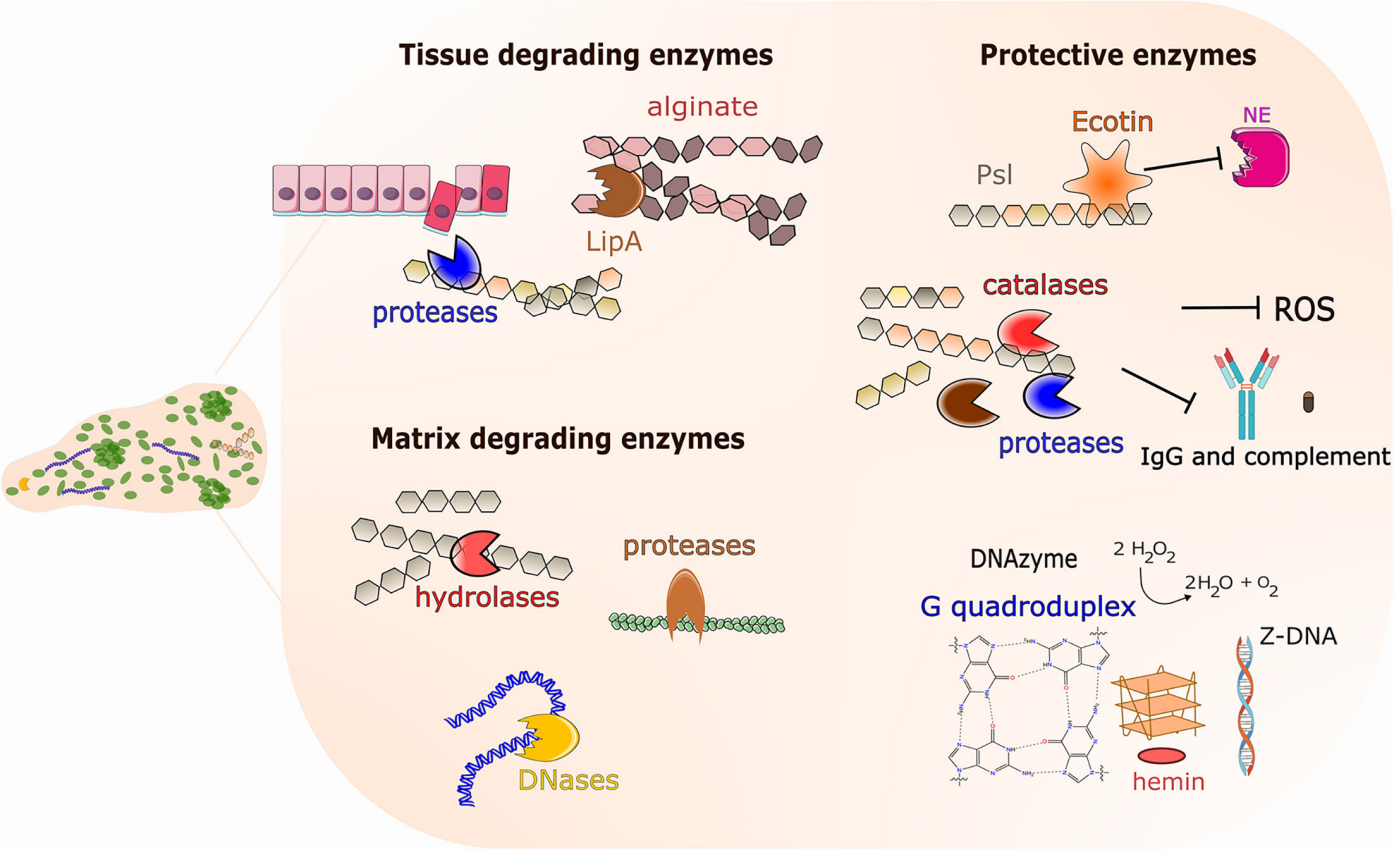
Catalytic properties of the extracellular matrix play a role in medically relevant biofilms. Matrix-associated protective enzymes shield the biofilm against host defences. Catalases and DNAzymes with peroxidase activity catalyse the reduction of reactive oxygen species from host neutrophiles, while Ecotin inhibits the activity of neutrophil elastase (NE). Proteases can target host defence mechanisms by degrading IgG and serum complement components. Additionally, matrix-associated proteases and lipases facilitate host-tissue degradation for either nutrient acquisition or to further the infection. Proteases, hydrolases and DNases released into the matrix through secretion or cell lysis help remodel or degrade the matrix.

Evidence is accumulating that the matrix of medically relevant biofilms has various catalytic functions (Fig. 2). Research on this topic has mainly been done on *P. aeruginosa* biofilms, and recently also on *Staphylococcus epidermidis* biofilms. Biofilms of these organisms are involved in various soft tissue and catheter/implant-associated infections. *P. aeruginosa* is generally capable of producing biofilm matrix components such as eDNA, the exopolysaccharides alginate, Psl and Pel, and proteins such as CdrA, Cup, LecAB and type IV pili [1]. Most *S. epidermidis* strains are capable of producing biofilm matrix components such as eDNA, *N*-acetylglucosamine (PNAG) and various cell wall-anchored proteins (e.g. Embp) [10].

Toyofuku *et al.* [11] conducted a comprehensive proteomic study to identify proteins associated with the biofilm matrix of * P. aeruginosa*. Several exoenzymes involved in macromolecule degradation were detected in the biofilm matrix. These were the proteases LasA (PA1871), LasB (PA3724), Protease IV (PA4175), alkaline protease (PA1249) and PasP (PA0423), as well as Chitinase (PA2300) and a probable aminopeptidase (PA2939). In addition, proteins related to oxidative stress were found in the biofilm matrix, for example the catalases KatA (PA4236) and KatE (PA2147) and the peroxidase PA3529. It is likely that some of these enzymes catalyse degradation of host tissue during infection, and that the oxidative stress-related enzymes protect against oxidative radicals generated by the neutrophils of the host immune system. Another protein, PA2959, identified in the biofilm matrix, is a putative DNase, which might play a role in biofilm dispersal by degrading matrix eDNA. Unlike the exoenzymes mentioned above, the oxidative stress-related proteins and the DNase are not secreted by the bacteria, and it is likely that they originate from lysed bacteria or membrane vesicles.

Tielen *et al*. [12] found that an extracellular lipase, LipA, produced by *P. aeruginosa* binds to alginate in the *P. aeruginosa* biofilm matrix. Thereby the lipase is immobilized and accumulates in the matrix near the bacterial cells. LipA was shown to be active when bound to alginate, and it was suggested that the presence of the lipase in the matrix may facilitate the uptake of fatty acids released from lipids by the action of the enzyme. Moreover, evidence was presented that binding of LipA to alginate protects the enzyme from denaturation and proteolytic degradation. Alginate is important in a clinical context, since a high proportion of *P. aeruginosa* strains isolated from the lungs of people with cystic fibrosis carry mutations so that they overproduce this exopolysaccharide.

Tseng *et al.* [13] used a biotinylation-based proteomic approach to identify matrix proteins in *P. aeruginosa* biofilms. The procedure may have resulted in a large amount of false negatives since known matrix proteins such as CdrA were not detected. Nevertheless, 60 proteins were identified to be present in the *P. aeruginosa* biofilm matrix. On the basis of their predicted biochemical activities, 19 of these 60 proteins were suggested to play a role in the protection of the biofilm. Among these 19 proteins, 12 were putative oxidoreductases, four were predicted to play a role in redox processes, two were predicted to facilitate protein folding and one, termed Ecotin (PA2755), is a putative serine protease inhibitor. Ecotin was enriched approximately fourfold in the matrix proteome, and it was shown to bind to Psl in the *P. aeruginosa* biofilm matrix. Matrix-associated Ecotin could inhibit neutrophil elastase, and it was demonstrated that Ecotin in the biofilm matrix could protect not only other matrix proteins but also biofilm cells from proteolytic attack. Since the protein sequence of Ecotin indicates that it is not secreted by the bacteria, it was suggested that it is released via lysis of a fraction of the bacteria during biofilm development.

Recently, evidence was provided that the catalytic functions of the biofilm matrix is not restricted to its proteins, but also is a feature of its eDNA. Minero *et al.* [10] showed that non-canonical DNA structures form in eDNA-rich staphylococcal biofilms, and that these structures might protect the biofilm from degradation by nucleases and the activity of peroxides. The observations were done in *S. epidermidis* biofilms grown in laboratory medium supplemented with haemin and NaCl to stabilize secondary DNA structures. Z-DNA and G-quadruplex DNA were demonstrated to be abundant in the biofilm matrix. Z-DNA is left-handed unlike the normal right-handed B-form of DNA. G-quadruplex DNA has a helical shape and contains guanine tetrads composed of one, two or four DNA strands. Mammalian DNase I lacked activity against Z-DNA and G-quadruplex DNA, but micrococcal nuclease could degrade G-quadruplex DNA, and S1 *Aspergillus* nuclease could degrade Z-DNA, suggesting that the latter enzymes might be useful for treatment of biofilm-related infections. In the presence of haemin, the G-quadruplex DNA in the biofilm matrix was shown to form a DNAzyme with peroxidase activity. The peroxidase activity of the matrix might protect infectious biofilms from hydrogen peroxide released from immune cells during the infection. G-quadruplex DNA and Z-DNA structures were also found in *Staphylococcus aureus* biofilms on implants in a murine implant-associated osteomyelitis model.

Biofilm matrices can also contain catalytic proteins that are involved in biofilm dispersal processes. Microbes generally form biofilm as long as the conditions are adequate, and transition to a planktonic state via a dispersal process when the conditions are unfavourable for biofilm formation. The biofilm dispersal process can, depending on the microbial species, be triggered by factors such as nutrient or oxygen limitation or the accumulation of intrinsic factors or waste products. In the case of *P. aeruginosa*, biofilm dispersal has been linked to increased activity of the periplasmic protease LapG that cleaves the CdrA adhesin, as well as increased expression of genes encoding the endonuclease EndA that degrades eDNA, and the glycoside hydrolase PelA that degrades Pel [14].

## Concluding remarks

The catalytic functions of the extracellular matrix of environmental biofilms are essential for the degradation of complex biomolecules, pollutant remediation, electron transfer and the functioning of bioelectrochemical systems. By mediating these processes, the biofilm matrix contributes to carbon cycling and environmental sustainability and offers potential for innovative biotechnological applications. Understanding and harnessing these catalytic properties can lead to advancements in fields such as waste treatment, energy production and the synthesis of valuable biochemicals.

The matrix of medically relevant biofilms contains proteases and lipases that may degrade host tissue and provide lipids and amino acids to the bacteria. It can also contain serine protease inhibitors that may inhibit neutrophil elastase. In addition, the biofilm matrix may contain proteins with catalase and peroxidase activity that can defend against neutrophil-mediated oxidative stress. Moreover, the eDNA of the biofilm matrix can form DNAzymes with peroxidase activity that may aid in the defence against neutrophil-mediated oxidative stress. Polysaccharide hydrolases, proteases and DNases may also play a role in biofilm dispersal and the spread of infections. Knowledge of the catalytic functions of the matrix of infectious biofilms may help identify drug targets for the development of novel drugs to combat these problematic infections.

## Abbreviation

eDNA: extracellular DNA
